# Dissemination and genome characterization of a human adenovirus F41 in a patient with B-Cell lymphoma

**DOI:** 10.1186/s12985-023-02101-3

**Published:** 2023-07-06

**Authors:** Amary Fall, Victoria L. Campodónico, Craig Howard, Nicholas Gallagher, Gabrielle Bailey, Adriana E. Kajon, Heba H. Mostafa

**Affiliations:** 1 Hopkins Medicine, Department of Pathology, Medical Microbiology, Baltimore, MD USA; 2 Department of Pathology, Medical Microbiology, Johns Hopkins Hospital, Baltimore, MD USA; 3 Lovelace Biomedical Research Institute, Albuquerque, NM USA

**Keywords:** Adenovirus F41, Dissemination, B-cell lymphoma, Genome characterization

## Abstract

Adenovirus (HAdV) F41 is a common cause of gastroenteritis and has rarely been reported associated with disseminated disease. In this report, an adult patient with a history of ulcerative colitis, cryptogenic cirrhosis, stage III adenocarcinoma, high-grade diffuse large B-cell lymphoma on chemotherapy was diagnosed with disseminated adenovirus infection. HAdV DNA was quantified in stool, plasma, and urine with viral loads of 7, 4, and 3 log10 copies/mL, respectively. The patient’s course was rapidly progressive and he passed away 2 days after initiation of antiviral therapy. The patient’s infecting virus was characterized as HAdV-F41 by whole genome sequencing.

## Background

Human adenoviruses (HAdV) are double-stranded nonenveloped DNA viruses that belong to the family *Adenoviridae*, genus *Mastadenovirus* [1]. More than 100 genotypes are classified within seven species designated HAdV-A through -G [2, 3]. HAdVs can cause a wide spectrum of diseases, including respiratory illness of variable severity, gastroenteritis, and conjunctivitis [4, 5]. Disseminated disease can occur in allogeneic hematopoietic stem cell transplant (HSCT) or cord-blood transplant patients [5, 6], h and has been described in other immunocompromised patients such as those on chemotherapy [4, 7–9], solid organ transplant recipients [10], individuals living with human immunodeficiency virus [11], premature infants [12, 13], and very rarely in immunocompetent adults [14–16]. Disseminated disease is more commonly associated with HAdV types A31, C1, C2, C5 and B21 [10, 17], and cases involving other types have also been described [18].

HAdV-F41, is one of the two enteric types of species HAdV-F. Like type F40, it is highly endemic globally, circulates year-round and causes gastroenteritis in children [19–21]. Considerable intratypic genetic variability has been described for HAdV-F41 with two major clades identifiable by phylogenetic analysis of whole or partial genomic sequences [22].

Although rare, disseminated HAdV-F41 disease has been reported in a 5-month-old child in the context of severe acute graft-versus-host disease after HSCT [23] and, in association with Epstein-Barr virus (EBV) encephalitis, in a teenage boy with acute lymphoblastic leukemia [24]. Interestingly, HAdV-F41 has been recently detected in immunocompetent children presenting with acute hepatitis, although the significance of these findings is debatable [25].

Here we describe a case of disseminated HAdV F41 disease in an adult patient with high-grade diffuse large B-cell lymphoma without history of HSCT.

## Results

The patient was an adult male with history of ulcerative colitis, and a partial bowel resection secondary to a perforated appendix. He was diagnosed with cryptogenic cirrhosis and several episodes of encephalopathy leading to hospitalization and with stage III adenocarcinoma on the right side of the colon followed by hemicolectomy and ostomy placement. The patient presented with an enlarging neck mass, was diagnosed with high-grade diffuse large B-cell lymphoma, and started RCHOP (rituximab, cyclophosphamide, doxorubicin, vincristine, prednisone) chemotherapy a month later.

After starting his second cycle of chemotherapy, the patient presented to the emergency department with rigors, dizziness, increased watery ostomy output, decreased appetite, decreased urine output and one episode of emesis. He was afebrile and hemodynamically stable. The laboratory work-up showed leukocytosis (he had received G-CSF (filgrastim) prior to admission), 26,420 WBC/mL(90% neutrophils), and thrombocytopenia (140,000 platelets/mL), acute kidney injury, elevated lactate (2.7 mmol/L), D-Dimer (3.25 mg/L) and lipase (904 UL) and moderate microscopic hematuria. Blood cultures were drawn and antibiotic treatment with intravenous cefepime was started. CT of the chest/abdomen/pelvis did not show evidence of acute abnormalities.

Two days after admission, his laboratory work-up showed increased leukocytosis (91,010/mL), anemia (Hgb 10.2 g/dL) and thrombocytopenia (73,000 platelets/mL), continued acute kidney injury with metabolic acidosis and signs of hepatitis (total bilirubin 4.7 mg/dL, AST 43 U/L, ALT 41 U/L, alkaline phosphatase 156 U/L, albumin 2.6 g/dL and total protein 4.9 g/dL). The patient remained stable with on and off vomiting and increased ostomy output (3 L/day). His stool *C. difficile*, norovirus and cytomegalovirus tests were negative.

On day five after admission, the patient showed episodes of atrial fibrillation with rapid ventricular response and hypotension, and signs of gastrointestinal bleeding with melena output through his ostomy and worsening coagulopathy (INR 1.9, prothrombin time 48.7 s, fibrinogen < 35 mg/dL), thrombocytopenia (37,000 platelets/mL) and hepatic function (bilirubin (8.8 mg/dL) and alkaline phosphatase (183 U/L)). The patient underwent an esophagogastroduodenoscopy and an ileoscopy which showed diffuse friable mucosa throughout the duodenum and small bowel with areas of spontaneous oozing of fresh blood with no identifiable lesion to explain bleeding.

Seven days after admission, the patient was diagnosed with disseminated adenovirus infection by a positive plasma PCR with a viral load of 109,410 copies/ mL (Log value 5.04). Adenovirus DNA was also detected in stool and urine specimens by real-time PCR. Digital droplet PCR (ddPCR) was used for HAdV quantification in plasma, urine and stool using a research use only assay which showed highest viral load in the stool sample, as previously described (Table 1) [26]. The patient was started on cidofovir and intravenous immunoglobulin. However, he developed altered mental status, hypothermia, hypotension and leukopenia (WBC 60/mL) requiring transfer to the ICU and administration of vasopressor support without showing signs of improvement. The patient was transitioned to comfort care and passed away 2 days after initiation of antiviral therapy.

**Table 1:** Adenovirus load in clinical specimens by ddPCR

Specimen	Log DNA copies/mL
Plasma	4
Urine	3
Stool	7

Other negative workup included serological testing for HSV-1/2, VZV, hepatitis B and C, and EBV, in addition to Histoplasma and Cryptococcus antigen testing and stool protozoan molecular and microscopic testing. No post-mortem investigations were performed. Hence, the disseminated adenovirus infection was regarded as the cause of the patient’s multi-organ failure and death.

**Fig. 1:**
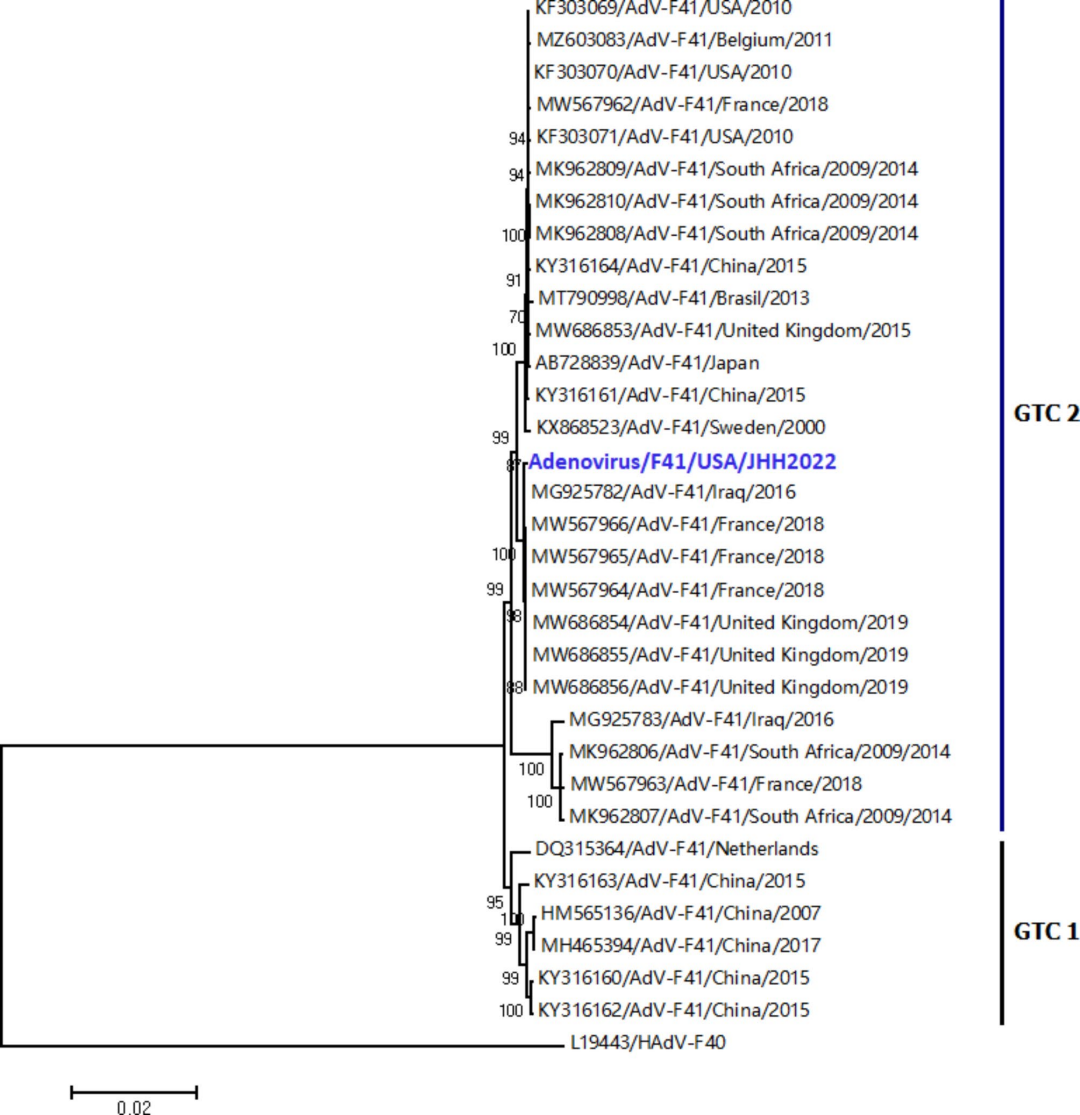
The phylogenetic tree was generated using a Maximum likelihood algorithm with the Kimura 2-parameter model nucleotide substitution model using MEGA 7 to estimate the evolutionary distances. The statistical significance was tested by 1000 bootstrapping replicates. Bootstrap values > 70% are shown at the branch nodes. The whole genome sequence obtained in this study is colored blue and the complete genome sequences of the HAdV-F41 available in GenBank are colored black. The tree is rooted by the HAdV-F40.

Whole genome sequencing was performed from total DNA extracted from stool and plasma specimens following non-targeted random amplification using the REPLI-g WGA & WTA kit (Qiagen). Library preparation was carried out using the Illumina DNA Prep kit (Illumina, United States) according to the manufacturer’s protocol. BLAST analysis (NCBI) of the consensus genomic sequence obtained identified the detected HAdV as type HAdV-F41. The complete genome sequence (GenBank accession number ON713486) was aligned with HAdV-F40 (outgroup) and with 32 HAdV-F41 whole genome sequences available in GenBank with MAFFT software (7.450). A maximum-likelihood tree was constructed using MEGA software version 7 based on the Kimura 2-parameter model (bootstraps = 1,000). The phylogenetic analysis revealed that the patient’s HAdV-F41 belonged to the clade GTC 2 and was closely related to strains circulating in Iraq, France, and United Kingdom in 2016, 2018 and 2019, respectively (Fig. 1). The genomic sequence shared 99.83% and 99.81% homology with strains detected in Iraq (MG925782) and France (MW567966), in 2016 and 2018 respectively, the two most closely related genomes.

## Discussion

Although gastroenteritis associated with HAdVF41 is highly prevalent and can be severe in infants, especially during outbreaks in pediatric bone marrow transplant units [27, 28], disseminated disease is extremely rare [23, 24, 27, 29]. In this study, we report a case of enteric infection with HAdV-F41 resulting in a disseminated fatal disease in an immunocompromised patient. Viral DNA was readily detected in plasma, urine, and in high concentrations in stool.

HAdVs, particularly species HAdV-C, -A, -B and -E [3, 30], can establish a persistent infection. The gastrointestinal tract is an important site for persistence and shedding of the virus into the stool after reactivation occurs [3]. Therefore, adenoviral infections detected in immunocompromised patients can result from exogenous acquisition or from reactivation [31]. In our patient, the presence of HAdV in the stool was not evaluated prior to initiation of chemotherapy or start of gastrointestinal symptoms. Therefore, we cannot conclude whether his infection represents an endogenous or an exogenous infection. However, since the highest viral load was detected in the stool and was identified as HAdV-F41 by whole genome sequencing, it would be reasonable to conclude that the infection originated in the gastrointestinal tract, especially in view of the patient’s initial presentation with diarrhea and dehydration. The HAdV-F41 detected in this study has a similarity of 99.8% per blast with strains that have previously been identified as being associated with an outbreak in pediatric hematopoietic stem cell transplant recipients with two fatal cases in France [28]. Although dissemination of HAdV-F41 is not common, clinicians should be aware that it can happen even in immunocompromised adult patients without history of HSCT.

## Data Availability

Sequence is available in GenBank under accession number of ON713486.
